# Nerve growth factor in combination with Oxiracetam in the treatment of Hypertensive Cerebral Hemorrhage

**DOI:** 10.12669/pjms.341.13395

**Published:** 2018

**Authors:** Yuzhen Sun, Baoquan Xu, Qiang Zhang

**Affiliations:** 1 Yuzhen Sun, Department of Neurosurgery, Binzhou People's Hospital, Shandong, 256600, China; 2 Baoquan Xu, Department of Neurosurgery, Binzhou People's Hospital, Shandong, 256600, China; 3 Qiang Zhang, Department of Neurosurgery, Binzhou People's Hospital, Shandong, 256600, China

**Keywords:** Hypertensive cerebral hemorrhage, Nerve growth factor, Oxiracetam

## Abstract

**Objective:**

To compare the clinical efficacy of nerve growth factor (NGF) in combination with oxiracetam and single use of oxiracetam in the treatment of hypertensive cerebral hemorrhage.

**Methods:**

One hundred and forty patients with hypertensive cerebral hemorrhage who were admitted to the hospital from July 2015 to September 2016 were selected as research subjects and randomly divided into a treatment group which was treated by NGF in combination with oxiracetam and a control group which was treated by oxiracetam only. The clinical efficacy was observed, and the death of both groups was recorded.

**Results:**

The National Institutes of Health Stroke Scale (NIHSS) score, Glasgow Coma Scale (GCS) score and limbs muscle force of both groups improved after treatment, and the improvement of the treatment was superior to that of the control group, suggesting a significant difference (P<0.05). The reduction of serum inflammatory factor level of the treatment group was much larger than that of the control group after treatment, and the difference had statistical significance (P<0.05). The survival analysis suggested that the survival rates of the two groups had a statistically significant difference (P<0.05).

**Conclusion:**

NGF in combination with oxiracetam is significantly effective in treating hypertensive cerebral hemorrhage as it can apparently recover neurologic impairment and limbs muscle force. The therapy has important clinical application values.

## INTRODUCTION

Hypertensive cerebral hemorrhage refers to cerebral parenchymatous hemorrhage induced by the rupture of arteriole in the brain on the basis of hypertension, which is featured by high fatality rate, high disability rate, rapid onset and serious disease condition. There would be severe sequela even when patients survived from hypertensive cerebral hemorrhage. It severely affects the living quality of patients and also brings heavy burden to the society.^1^-^3^ Currently, the main therapies for hypertensive cerebral hemorrhage include conservative treatment, surgery and minimally invasive hematoma evacuation; however, those therapies have certain limitations in practical application, especially in recovering neurological function.^4^,^5^ The clinical efficacy of neuroprotective drugs in recovering neurological impairment of patients with stroke is always the hotspot in clinics. Oxiracetam as a neurotrophic drug is usually used for treating brain function degradation diseases and injury induced neurological deficit.^6^

It has been pointed out that^7^, single use of oxiracetam is not so effective in recovering neurocognitive function. But nerve growth factor (NGF) as an extensively applied neurotrophic factor (NTF) has been found playing an important role in the survival, growth, differentiation and regeneration of neurons.^8^,^9^ Currently, NGF has been extensively applied in treating cerebral infarction and spinal and peripheral nerve injury, but few studies are available concerning the clinical efficacy of NGF in treating intracranial hemorrhage. This study selected patients with hypertensive cerebral hemorrhage as the research subjects and investigated the clinical efficacy of NGF in combination with oxiracetam in the treatment of hypertensive cerebral hemorrhage and its influence on the levels of serum inflammatory factors high-sensitivity C-reactive protein (hs-CRP), interleukin (IL)-8 and tumor necrosis factor (TNF)-α.

## METHODS

One hundred and forty patients with hypertensive cerebral hemorrhage who were admitted to our hospital from July 2015 to September 2016 were selected. Patients who satisfied the criteria of hypertensive cerebral hemorrhage formulated in the 4^th^ National Conference of Cerebrovascular Disease, have been verified by CT, were admitted to the hospital within 48 hours after onset, were scored as 8 points higher by Glasgow Coma Scale (GCS), and were examined having normal hepatic and renal function were included. Those who had vascular malformation, chronic disease or disturbances of blood coagulation, had blood flowing into subarachnoid space or ventricle, whose GCS score no higher than three points, had symptoms such as bilateral pupils dilatation, disappearance of light reflex and severe disorder of vital signs, or had mental disorder or nerve dysfunction including impairment of nervous system function induced by other existing systemic diseases such as peripheral neuropathy previously were excluded.

This study was carried out after the patients and their family members signed informed consent and it was reviewed and approved by the ethics committee. All the patients were divided into a treatment group and a control group using random number table, 70 in each group. In the treatment group, there were 38 males and 32 females, with an average age of (68.5±4.8) years (50~83 years), average blood loss of (23.5±2.2) ml (10~31 mL) and average onset-to-door time of (5.4±1.5) h (2~36 hour); as to the bleeding sites, there were 44 cases of basal ganglia, 18 cases of blood brain and 8 cases of cerebral lobe. In the control group, there were 42 males and 28 females, with an average age of (69.2±5.3) years (51~82 years), an average blood loss of (23.2±1.9) ml (11~32 mL) and an average onset-to-door time of (5.8±0.5) hour (2~38 hour); as to the bleeding sites, there were 45 cases of basal ganglia, 16 cases of blood brain and 9 cases of cerebral lobe. Baseline data such as gender, age, blood loss and bleeding sites of the two groups had no statistically significant difference (P>0.05); the results were comparable.

### Treatment methods

Patients in the two groups were given continuous oxygen inhalation in a low or medium flow. Moreover, they were given relevant treatment such as blood pressure and glucose control, intracranial pressure reduction, brain cells nourishing and hemostasis. Water and electrolyte were kept stable and balanced, enough nutritional supply was provided, and infection was carefully prevented.

In addition to conventional treatment, each patient in the treatment group were intramuscularly injected with the liquid containing 30 μg of NGF (Weiming Biomedical Co., Ltd., China) and 2 mL of 0.9% sodium chloride injection, once a day. Moreover, 5 g of oxiracetam injection which was dissolved by 0.9% sodium chloride injection was intravenously injected, once a day, for four weeks.

In addition to conventional treatment, each patient in the control group was intravenously dripped with 5 g of oxiracetam (Oulaining, Ouyi Pharmaceutical Co., Ltd. of China Shijiazhuang Pharmaceutical Group) which was dissolved with 0.9% sodium chloride injection, once a day, for four weeks.

### Nursing after drug administration

Patients in the two groups were given effective nursing during drug administration. The nursing content was as follows. The first content was respiratory tract nursing. The patients were given turnover and backslap nursing, once every two hours. Respiratory secretions were timely removed to ensure smooth respiration and reduce risks of pulmonary infection. Next was nursing of vital signs. The respiration, blood pressure, heart rate, blood oxygenation concentration and body temperature were closely monitored. Patients whose temperature was higher than 38°C were given ice compress on the occiput or great vessels of four limbs; ice blanket or drugs could be used if necessary. The consciousness and pupils of the patients were observed, hourly; the critically ill patients were observed once every 30 minutes. The disturbance of consciousness was evaluated. Once symptoms of increased intracranial pressure such as abnormal diameter of pupils, weakened pulse, increase of blood pressure and emesis were observed, therapies for lowering intracranial pressure and relieving dehydration were adopted to prevent the occurrence of cerebral hernia. The third was nursing of drainage tube. Drainage tubes were properly fixed and labeled clearly. The property, amount and color of the drainage liquid and whether the drainage tubes were smooth were closely observed. The height of the drainage tubes was adjusted according to the disease conditions of the patients or the amount of drainage liquid. The excessively high position of drainage tubes might lead to inadequate drainage, while the excessively low position might accelerate drainage and even induce cerebral hernia. The drainage tubes should be removed after one week to reduce the incidence of intracranial infection. Moreover, sterile operation and regular air sterilization should be strictly performed. Last was nursing of diet. Food with high protein, low fat, high heat and high cellulose were recommended for the patients. Moreover, the intake of sodium salt should be limited, and the temperature of food should be proper. Those who could not take food by themselves because of coma were provided liquid food such as soybean milk, milk, steamed eggs, lotus root starch and evenly mixed pasty food by means of nasal feeding, four to five times each day, 200 ~ 300 ml/L each time.

### Observation indicators

NIHSS score,^10^ muscle strength grade and GCS score were taken as the evaluation indicators for clinical efficacy. The evaluation was performed once before and after drug administration to understand the improvement of neurological function. 2 mL~4 mL of fasting venous blood was collected from each patient in the morning and centrifuged at 3000 r/min at 4°C for 10 min. The supernate was collected and preserved at -60°C. The serum high-sensitivity C-reactive protein (hs-CRP) level was detected using double antibody enzyme linked immunosorbent assay. The levels of serum interleukin (IL)-8 and tumor necrosis factor (TNF)-α were detected using radioimmunoassay. The patients were followed by telephone interview assisted by visit or admission for 14 months. The survival condition of the patients was observed as well.

### Statistical analysis

Data were statistically analyzed by SPSS. ver. 21.0. Measurement data were expressed as mean±standard deviation (SD). The comparison between the two groups was performed by t test. Enumeration data were processed by Chi-square test. Survival analysis was estimated by Kaplan-Meier method and tested by Log-rank test. Difference was considered as statistically significant if P<0.05.

## RESULTS

### Recovery of neurological function

Before treatment, the NIHSS score, GCS score and muscle strength grade of the two groups showed no significant difference (P>0.05). After treatment, the NIHSS score, GCS score and muscle strength grade of the two groups improved, and the improvement of the treatment group was more obvious than that of the control group, suggesting a statistically significant difference (P<0.05; Table-I).

**Table-I:** Comparison of NIHSS score, GCS score and muscle strength grade between the two groups before and after treatment.

Group		NIHSS score	GCS score	Muscle strength grade
Treatment group (N=70)	Before	11.43±3.61	8.45±3.48	2.12±0.71
	After	6.90±2.93*	13.16±3.57*	4.84±0.79*
Control group (N=70)	Before	10.32±3.48	8.36±3.87	1.93±0.87
	After	8.13±2.92	9.17±4.05	3.36±0.41

* *Note:* indicated P<0.05 compared to the control group.

### Elimination of serum inflammatory factors

The difference of the levels of serum inflammatory factors hs-CRP, IL-8 and TNF-α between the two groups had no statistical significance before treatment (P>0.05). After treatment, the levels of hs-CRP, IL-8 and TNF-α of the treatment group decreased obviously (P<0.05), and the decrease was more obvious than that in the control group (P<0.05; Table-II).

**Table-II:** Comparison of hs-CRP, IL-8 and TNF-α between the two groups before and after treatment.

Group		hs-CRP (mg/L)	IL-8 (ng/L)	TNF-α (ng/L)
Treatment group (N=70)	Before	42.03±7.83	21.03±5.11	22.12±3.77
	After	18.02±4.01*	7.78±2.55*	7.24±3.18*
Control group (N=70)	Before	41.97±8.01	20.35±4.83	21.18±3.51
	After	30.97±6.02	14.31±3.18	14.47±2.86

* *Note:* indicated P<0.05 compared to the control group.

### Survival analysis

**Fig. 1:**
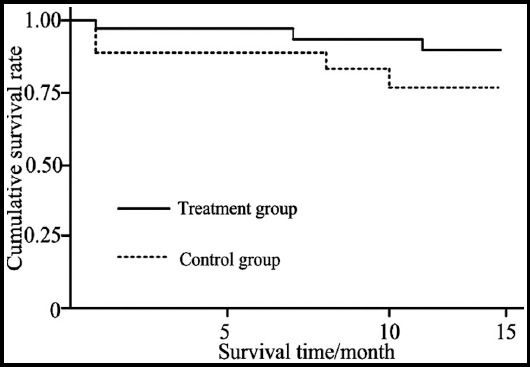
The survival conditions of patients in the two groups in the follow-up period.

In the whole follow-up period, three patients in the treatment group died, one in the 1^st^ month after treatment, one in the 7^th^ month after treatment and one in the 11^th^ month after treatment; fourteen patients in the control group died, seven in the 4^th^ month after treatment, two in the 8^th^ month after treatment, one in the 9^th^ month after treatment and four in the 13^th^ month after treatment. The Kaplan-Meier survival curves of the two groups are shown in Fig.1. The Log-rank test suggested the two groups had statistical significance (P<0.05).

## DISCUSSION

Patients with hypertensive cerebral hemorrhage may have pathological changes such as cerebral tissue injury, neuron injury and inflammatory reaction, which can accelerate the death of brain cells and disorder of brain cognitive function.^11^ Oxiracetam, a neurotrophic drug which can promote the abnormality of central nervous system, can regulate excitement of nervous system, inhibiting transmission of neural signals and neurohormones, and affect cognitive and neurological function by directly acting on neuroceptors.^12^ Moreover, oxiracetam can accelerate the recovery of function of nerve cells by affecting the energy metabolism of nervous system. But a clinical study found that single use of oxiracetam may be limited in recovering neurological function of patients with hypophrenia or Alzheimer's disease.^7^

NGF, the neuroactive factor which was discovered earliest, plays a key role in the growth and phenotype maintenance of peripheral nerves as well as the complexity of cholinergic neurons in central nervous system.^13^ NGF has been extensively applied in treating many diseases such as diabetic peripheral neuropathy, acute spinal cord injury and injury of optic nerve.^14^,^15^ The research results suggested that the treatment group which were treated by oxiracetam in combination with NGF obtained significant treatment effect; the indicators for the recovery of neurological function of the treatment group were superior to those of the control group, which further suggested that NGF in combination with oxiracetam was effective in recovering neurological function. That was because that NGF could protect nerve cells, inhibit cerebral injury induced neuronal death, promote the growth of nerve cells and the proliferation of neurogliocyte, and increase the communication between synapse.^16^

In recent years, the role of inflammatory reactions in the occurrence and development of cerebral hemorrhage has been more and clearer. The formation of hematoma after the occurrence of acute cerebral hemorrhage can promote the release of a large amount of inflammatory factors and inflammatory mediators which participate in secondary brain injury that is the key factor influencing the prognosis of patients.^17^ TNF-α, IL-8 and hs-CRP as the important regulatory factors in inflammatory reaction and immune response plays important roles in the occurrence of secondary brain injury induced by intracranial hemorrhage.^18^ Therefore, levels of TNF-α, IL-8 and hs-CRP can reflect edema around intracranial hematoma of patients with intracranial hemorrhage, which is of great significance to studies on the treatment of acute cerebral hemorrhage. In this study, the reduction of levels of hs-CRP, TNF-α and IL-8 of the treatment group was more obvious than that of the control group, indicating the combination therapy had stronger inhibitory effect on the serum cytokines than single use of drug, indicating NGF could reduce inflammatory reaction, weaken peroxidatic reaction and relieve neuron damage. A study found that timely neuroprotection had a great effect on the prognosis of patients.^19^ The survival analysis suggested that the survival rate of the treatment group was much higher than that of the control group, suggesting NGF was helpful to improve the long-term treatment efficacy of patients with hypertensive cerebral hemorrhage.

## CONCLUSION

In conclusion, NGF in combination with oxiracetam has significant effect in the treatment of hypertensive cerebral hemorrhage as it can effectively reduce the levels of inflammatory factors, improve neurological function and enhance living quality. The therapy is worth clinical promotion. But the medication safety has not been considered. Therefore, multi-center clinical experiments involving a large size of samples should be carried out in the future to investigate the adverse reactions occurring in the treatment of hypertensive cerebral hemorrhage with NGF in combination with oxiracetam.

### Authors' Contribution

**YZS & QZ:** Study design, data collection and analysis.

**YZS, BQX & QZ:** Manuscript preparation, drafting and revising.

**YZS:** Review and final approval of manuscript.
